# Carbon Source-Dependent Changes of the Structure of *Streptococcus pneumoniae* Capsular Polysaccharide with Serotype 6F

**DOI:** 10.3390/ijms22094580

**Published:** 2021-04-27

**Authors:** Joel P. Werren, Lukas J. Troxler, Oluwaseun Rume-Abiola Oyewole, Alban Ramette, Silvio D. Brugger, Rémy Bruggmann, Mark van der Linden, Moon H. Nahm, Ilche Gjuroski, Carlo Casanova, Julien Furrer, Markus Hilty

**Affiliations:** 1 Institute for Infectious Diseases, University of Bern, 3001 Bern, Switzerland; joel.werren@ifik.unibe.ch (J.P.W.); ljtroxler@outlook.com (L.J.T.); oluwaseun.oyewole@ifik.unibe.ch (O.R.-A.O.); alban.ramette@ifik.unibe.ch (A.R.); carlo.casanova@ifik.unibe.ch (C.C.); 2 Graduate School for Cellular and Biomedical Sciences, University of Bern, 3001 Bern, Switzerland; 3 Department of Infectious Diseases and Hospital Epidemiology, University Hospital Zurich, University of Zurich, 8091 Zurich, Switzerland; Silvio.Brugger@usz.ch; 4 Interfaculty Bioinformatics Unit and Swiss Institute of Bioinformatics, University of Bern, 3001 Bern, Switzerland; remy.bruggmann@bioinformatics.unibe.ch; 5 German National Reference Centre for Streptococci, Department of Medical Microbiology, University Hospital RWTH Aachen, 52074 Aachen, Germany; mlinden@ukaachen.de; 6 Department of Medicine, University of Alabama at Birmingham, Birmingham, AL 35294, USA; mnahm@uabmc.edu; 7 Department of Chemistry and Biochemistry, University of Bern, 3001 Bern, Switzerland; ilche.gjuroski@dcb.unibe.ch (I.G.); julien.furrer@dcb.unibe.ch (J.F.)

**Keywords:** *Streptococcus pneumoniae*, capsular polysaccharide, nuclear magnetic resonance (NMR), serogroup 6, galactose, glucose, vaccines

## Abstract

The structure of the exopolysaccharide capsule of *Streptococcus pneumoniae* is defined by the genetic arrangement of the capsule operon allowing the unequivocal identification of the pneumococcal serotype. Here, we investigated the environment-dependent composition of the polysaccharide structure of *S. pneumoniae* serotype 6F. When grown in a chemically defined medium (CDM) with glucose versus galactose, the exopolysaccharide capsule of the serotype 6F strains reveals a ratio of 1/0.6 or 1/0.3 for galactose/glucose in the capsule by ^1^H-NMR analyses, respectively. Increased production of the capsule precursor UDP-glucose has been identified by ^31^P-NMR in CDM with glucose. Flow cytometric experiments using monoclonal antibodies showed decreased labelling of Hyp6AG4 (specific for serotype 6A) antibodies when 6F is grown in glucose as compared to galactose, which mirrors the 1H-NMR results. Whole-genome sequencing analyses of serotype 6F isolates suggested that the isolates evolved during two different events from serotype 6A during the time when the 13-valent pneumococcal conjugate vaccine (PCV-13) was introduced. In conclusion, this study shows differences in the capsular structure of serotype 6F strains using glucose as compared to galactose as the carbon source. Therefore, 6F strains may show slightly different polysaccharide composition while colonizing the human nasopharynx (galactose rich) as compared to invasive locations such as the blood (glucose rich).

## 1. Introduction

*S. pneumoniae* asymptomatically colonizes the human nasopharynx, but it can also invade different body niches and cause diverse diseases such as acute otitis media, bronchitis, sinusitis, (bacteremic) pneumonia, sepsis and meningitis [1,2]. The most important virulence factor of *S. pneumoniae* is the polysaccharide capsule and based on its biochemical composition, more than 100 pneumococcal serotypes have been defined [3]. The introduction of pneumococcal conjugate vaccines (PCVs) has largely decreased the incidence of invasive pneumococcal disease (IPD) but may not be the ultimate solution as serotype redistribution has taken place [4,5]. Currently, PCV10 and PCV13 are widely used [5,6] but there are intentions of introducing PCV15 and PCV20 in the near future [7,8].

Different body niches present pneumococci with different nutritional milieus. The respiratory tract contains a lot of mucins, which are glycoproteins rich in N-acetylglucosamine (GlcNAc), N-acetylgalactosamine (GalNAc), galactose and N-acetylneuraminic acid (NeuNAc). These carbohydrates are degraded by the glycan-specific metabolic machinery of *S. pneumoniae* [9]. In the blood, unlike the respiratory tract, glucose is the most abundant sugar, reflecting that *S. pneumoniae* meets niches with different nutritional environments, leading to alterations in the transcriptome [10].

**Figure 1:**
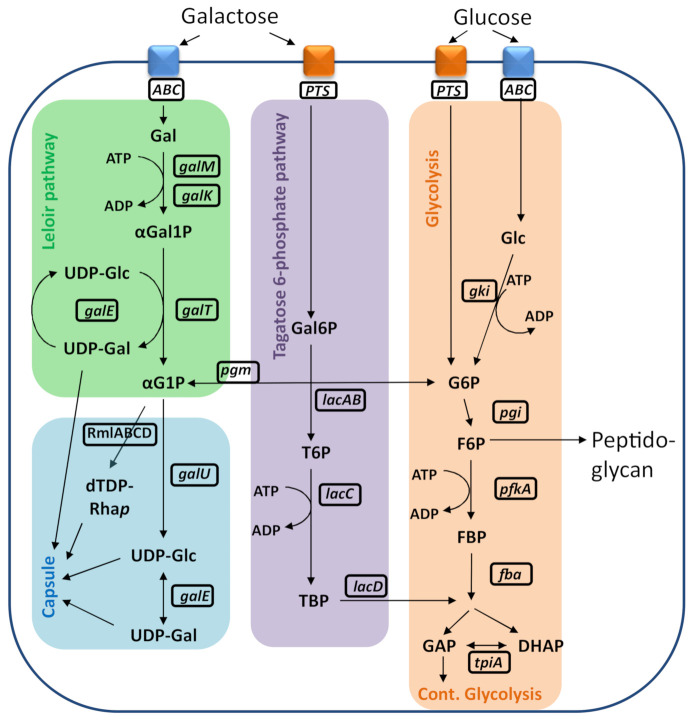
Schematic illustration of metabolic pathways in *S. pneumoniae*. DHAP: dihydroxyacetone phosphate; dTDP-Rha*p*; dTDP-L-rhamnose; *fba*: fructose-bisphosphate aldolase; F6P: fructose 6-phosphate; FBP: Fructose-1,6-bisphosphatase; *gale*: UDP-glucose 4-epimerase; *galK*, galactokinase *galM*, aldose 1-epimerase; *galT*, galactose 1-phosphate uridylyltransferase; Gal-6P: galactose 6-phosphate *galU*: glucose 1-phosphate uridylyltransferase; GAP: glyceraldehyde 3-phosphate; *gki:* glucokinase; Glc-1,5lac-6P: glucono-1,5-lactone 6-phosphate; Glc-1P: glucose 1-phosphate; Glc-6P: glucose 6-phosphate; *lacA*, galactose 6-phosphate isomerase subunit LacA; *lacB*, galactose 6-phosphate isomerase subunit *LacB*; *lacC*, tagatose 6-phosphate kinase; *lacD*, tagatose 1,6-diphosphate aldolase; *lacFE* galactose and lactose uptake phosphotransferase transporter; *manLMN*: monosaccharide uptake phosphotransferase transporter; *pfkA*: ATP-dependent 6-phosphofructokinase; *pgi*, glucose 6-phosphate isomerase; *pgm*: phosphoglucomutase; *PTS:* phosphotransferase transporter; *RmlA*, glucose-1-phosphate thymidylyltransferase; *RmlB*, dTDP-D-glucose 4,6-dehydratase; *RmlC*, dTDP-4-keto-6-deoxy-D-glucose 3,5-epimerase; *RmlD*, dTDP-4-keto-L-rhamnose reductase; T6P: tagatose-6-phosphate; TBP: tagatose 1,6-Bisphosphate; *tpiA*: triosephosphate isomerase; UDP-Gal: uridine diphosphate galactose; UDP-Glc: uridine diphosphate glucose; *RmlABCD* are specific capsule genes for serogroup 6 strains.

Glucose is imported by both a phosphoenolpyruvate-phosphotransferase system (PTS) (e.g., by *manLMN*) and an ABC transporter into the cell where it is metabolized via glycolysis (Figure 1) [11]. In contrast, galactose, which can be imported via PTS (e.g., *lacFE*) or the ABC transporters, is first metabolized via the tagatose or the Leloir pathway before entering glycolysis (Figure 1). Thus far, it is widely believed that the carbon source environment does not affect the structure of the polysaccharide capsule [12]. This is an important assumption for the accurate determination of pneumococcal serotypes in the diagnostic laboratories and for the correct design of pneumococcal conjugate vaccines. As an example, serotype 19A isolates from Switzerland showed no differences in composition or linkage of their subtype capsular saccharides despite genomic variations within the capsule operon [12]. Generally, isolates expressing identical serotypes may show large SNP differences in the capsule region/operon [13,14]. Therefore, hypothetical structural differences based on genomic variations should always be confirmed chemically in order to define new serotypes [15,16,17].

However, Oliver et al. performed a more in-depth chemical characterization of two atypical, serogroup 6 isolates from Germany. Sequencing of the capsular wciNα gene, encoding α-1,3-galactosyltransferase, revealed a single substitution that caused an amino acid change, A150T, and resulted in the new hybrid serotypes known as 6F and 6G [17]. As for the latter, a second point mutation was observed.

In this study, we aim to investigate structural changes in capsular composition of serotype 6F strains according to the different carbon sources glucose and galactose. We hypothesize that this could be relevant for *S. pneumoniae* colonization versus bacteremia as the nasopharynx is supposedly galactose rich and the blood, glucose rich [18]. In addition, we investigate the evolution of the serotype 6F within serogroup 6 strains, by using whole-genome sequencing and phylogenetic analysis.

## 2. Results

### 2.1. Bacterial Growth under Different Conditions for Serogroup 6 Strains

**Figure 2:**
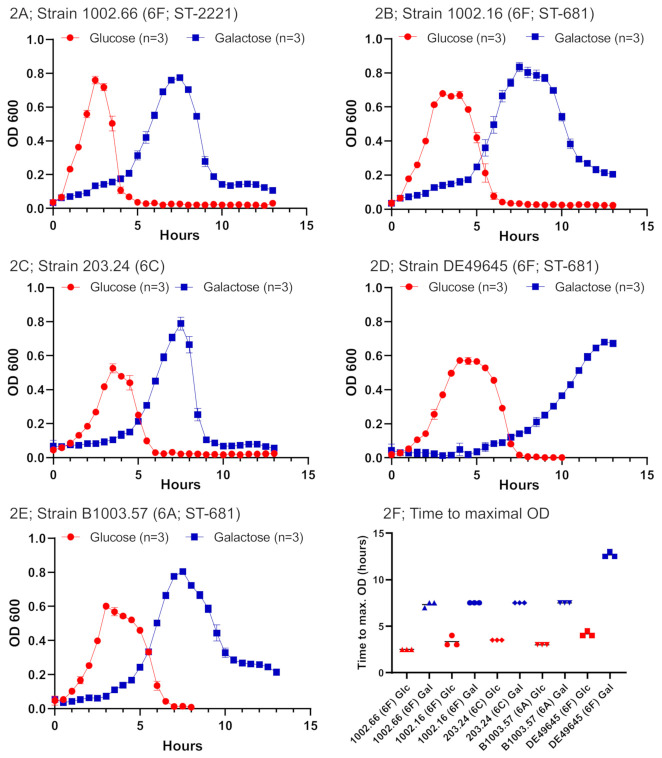
Growth curves of serogroup 6 strains. Growth measurement of serogroup 6 strains was performed in chemically defined media (CDM) with the indicated carbon sources glucose (red) or galactose (blue) (**A**–**E**). The times to max. (maximal) optical density (OD) are shown separately (**F**). ST; Sequence type.

From 2000 to 2018, more than 20,000 pneumococcal isolates from IPD cases were serotyped in Switzerland [6]. Within the collection, we discovered two isolates showing a result for serotype 6F, which has only been described for a single strain from Germany [17] (Table 1). The 6F strains contain a bispecific glycosyltransferase within the capsule operon and we therefore hypothesized that the capsule operon may not exclusively account for the capsular structure but that the polysaccharide capsule is also dependent on the available carbon sources. For testing this hypothesis, we first investigated whether the strains show different growth behavior. Serogroup 6 strains were grown in chemically defined medium (CDM) either containing glucose or galactose as carbon source to investigate the growth behavior. The uptake and metabolism of the two carbon sources have been previously investigated and are schematically illustrated in Figure 1 [18,19]. In our study, all strains showed similar growth behavior in CDM containing glucose (Figure 2A–E). In contrast, growth on galactose was delayed compared to glucose and showed differences between strains. In particular, the German 6F strain (DE49645) showed a very delayed growth as compared to the other strains. As for the latter, possible relevant single-nucleotide polymorphisms (SNPs) were observed after whole-genome sequencing and are shown and discussed below. The two 6F strains 1002.16 and 1002.66 showed similar growth on galactose. Overall, isolates grew faster on CDM containing glucose, compared to CDM with galactose.

### 2.2. NMR Capsule Structure Analysis Indicates Differences within 6F Strains According to the Used Carbon Sources

**Figure 3:**
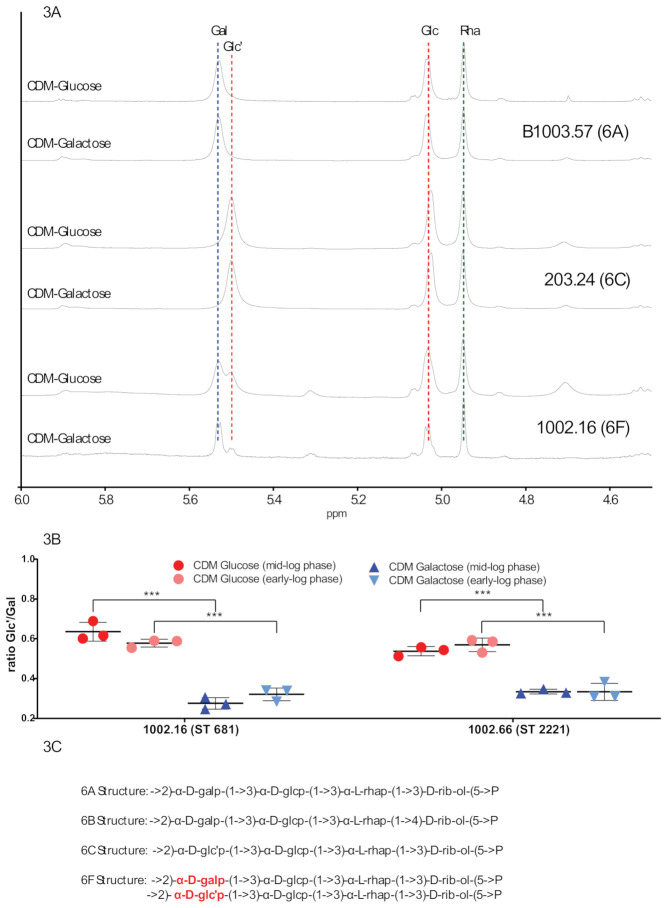
^1^H-NMR spectra of capsular polysaccharides of serogroup 6 strains. ^1^H-NMR spectra were obtained for different serogroup 6 strains grown in CDM with glucose (glc) or galactose (gal) (**A**). The ratio of 6C to 6A repeat unit incorporation measured by the integrals of the Glc’ and Gal resonances was calculated for all experiments (**B**). In total, we performed the measurements in triplicates at mid-log phase (OD_600nm_ = 0.4) and at early-log phase (OD_600nm_ = 0.25). Proposed structures for serogroup 6 strains are shown (**C**). Glc’ and Gal within 6F strains are indicated in red. *** indicates *p* < 0.001 (Unpaired *t*-tests).

We next aimed at investigating whether the polysaccharide composition differs when strains of serotype 6F are exposed to glucose as compared to galactose. To this purpose, the molecular structure of the capsular polysaccharide has been characterized by ^1^H-NMR spectroscopy and one-dimensional NMR data received (Supplementary Figure S1). Focusing on the anomeric signals in the ^1^H-NMR spectra, we confirmed that the capsule extracts of 1002.16 and 1002.66 demonstrated the presence of both type 6A and type 6C repeat units in the capsules of these strains (Figure 3A,B). This means that in addition to a glucose (Glc) and a rhamnose (Rha) monomer, the type 6A repeat unit also contained a galactose (Gal) monomer, while the type 6C unit contained an additional glucose (Glc’) (Figure 3C). While analyzing the type 6F spectra, we additionally observed a change in the ratio of sugar incorporation between the 6A and 6C repeat units under different nutritional conditions, as evidenced by the different sizes of the Gal and Glc’ peaks (Figure 3B), with a Glc’/Gal ratio of around 0.6/1 when grown on glucose and 0.35/1 when grown on galactose, respectively. In total, we performed the measurements in triplicates for the serotype 6F strains 1002.16 and 1002.66 at mid log phase (OD_600nm_ = 0.4). In addition, we performed the triplicate measurements at early-log phase (OD_600nm_ = 0.25) and again observed a significant decrease in the proportion of Glc’ incorporated when the strains were grown on galactose as the sole carbon source (Figure 3B). It has been previously suggested that ∼75% of 6F repeating units are identical to 6A, whereas 25% are 6C-like because they contain Glc’ [17]. We newly suggest that this proportion varies depending if Glucose or Galactose is the primary carbon source. We hypothesize that this phenomenon is due to the bispecific glycosyltransferase in type 6F strains preferentially introducing the more widely available sugar precursor into the capsule.

### 2.3. Analysis of Polysaccharide Capsule Metabolites by ^31^P NMR

**Figure 4:**
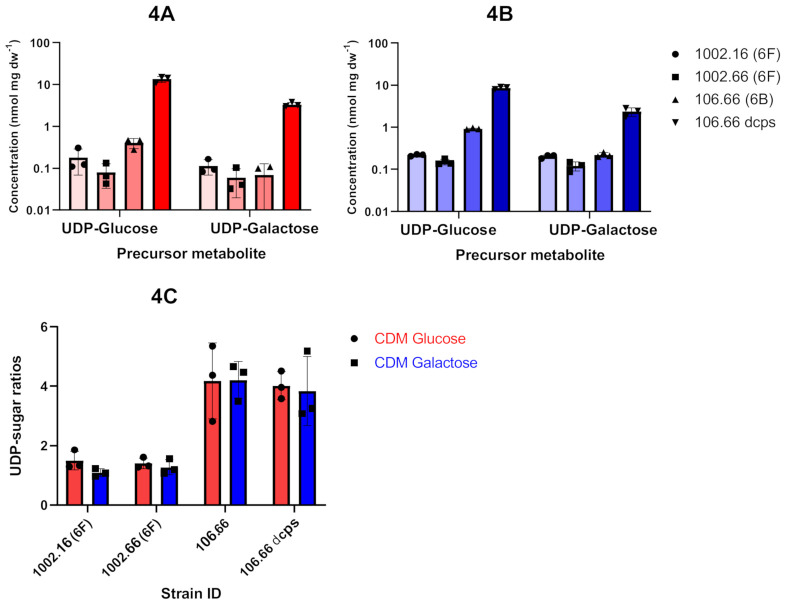
Capsule precursor levels observed in S. pneumoniae whole cell extracts. Overall absolute values for the precursors UDP-glucose and UDP-galactose are shown for the media with glucose (red; (**A**)) or galactose (blue; (**B**)). UDP-sugar rations are also shown (**C**). Values from three independent experiments are shown for the strains 1002.16 (serotype 6F), 1002.66 (serotype 6F), 106.66 (serotype 6B) and capsule knockout strain 106.66 (dcps).

We further hypothesized that the increased incorporation of glucose of serotype 6F strains during growth in glucose as opposed to galactose was due to an increase in the production of the precursor UDP-glucose as compared to UDP-galactose. We therefore compared the intracellular profiles of UDP-glucose and UDP-galactose of *S. pneumoniae* strains with serotype 6F in CDM supplemented with either glucose or galactose using ^31^P-NMR as previously described (Figure 4A,B) [21]. The experiments were also conducted for the wild-type and capsule knockout mutant of 106.66 (serotype 6B), respectively. Overall, the quantities of UDP-glucose and UDP-galactose were very low for the three wild-type strains with serotypes 6F and 6B. In contrast, capsule precursors clearly accumulated in the Δcps (knockout) strain of 106.66. We found an increased accumulation of UDP-glucose when grown in glucose as compared to galactose but the ratio of UDP-glucose/UDP-galactose remained unchanged (Figure 4C).

### 2.4. Flow Cytometry Analyses Show Different Bindings of Monoclonal Antibodies (mAbs)

**Figure 5:**
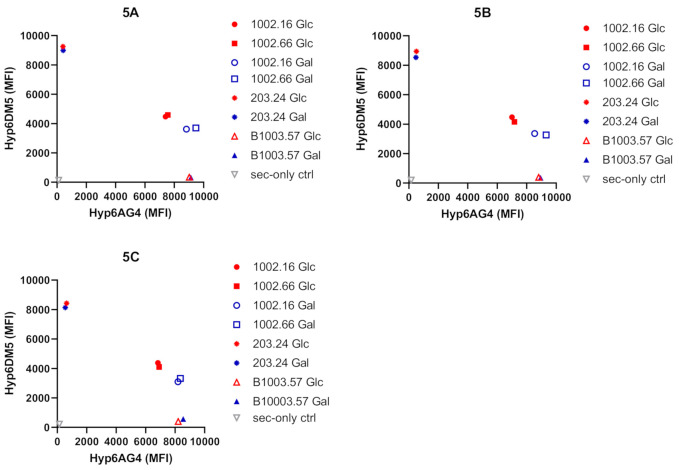
Comparison of immunologic properties of serogroup 6 strains. The strains with serotypes 6F (1002.16 and 1002.66), serotype 6A (B1003.57) and 6C (203.24) were investigated. For immunological analysis, all the strains were stained with a 6A (Hyp6AG4) or 6C (Hyp6DM5)-specific monoclonal antibodies (mAbs), the amounts of mAb bound to bacteria were determined with a flow cytometer, and the amounts (mean fluorescence intensity, MFI) were plotted in both axes. Figures are shown in three independent replicates. (**A**–**C**). Glc (glucose) and Gal (galactose) values are indicated in red and blue, respectively. Secondary-only stained control (sec-only ctrl).

The 6F strains were then studied for antigenic changes by flow cytometry using mAbs Hyp6AG4 and Hyp6DM5, which are specific for serotypes 6A and 6C, respectively. As expected, B1003.57 (6A) and 203.24 (6C) strains reacted with only one of the two mAbs, whereas the two serotype 6F strains reacted with both mAbs. Strikingly, different binding was detected for Hyp6AG4 and Hyp6DM5 when the 6F strains were grown in glucose as compared to galactose in three independent replicates (Unpaired *t*-tests; *p* < 0.001; Figure 5A–C). As expected from the 1H-NMR analyses, strains grown in glucose as compared to galactose showed a less serotype 6A-like binding behavior. Collectively, ^1^H-NMR and flow cytometry analyses indicated that 6F strains reveal a more serotype 6A like than 6C like capsular composition when grown in galactose.

### 2.5. Analysis of Genome Assemblies of Serogroup 6 Strains

The two Swiss and the German isolates were sequenced to better understand their evolutionary relationships. For this purpose, additional serotype 6A but not 6C isolates with identical sequence types (STs) were sequenced or, if available, sequences were derived from databases. Serotype 6C isolates were excluded as, unlike 6A and 6F isolates, they have a wciN*β* rather than wciN*α* allele in the capsule operon [17]. In total, 12 isolates were included in this analysis from the years 2003–2015 (Table 1). We identified several variable regions, of which nine were more prominent among the serotype 6F genomes (Supplementary Materials Figure S2; Supplementary Materials Table S1). Functional annotations were subsequently obtained for 89.9%, 64% and 55% of core, variable and unique genes, respectively (Supplementary Materials Table S2). Interestingly, SNPs within galactosamine-6-phosphate isomerase (AgaS) and within galactose-1-phosphate uridylyl transferase were identified for the strain DE49645 (Supplementary Materials Table S3) which could explain the delayed growth (Figure 2D). However, an explanatory role of the found SNPs remains a speculation as these mutations have not been further analyzed by functional genomics (by, e.g., creating null mutants) within this study.

### 2.6. Evolutionary Events Leading to Serotype 6F Strains

**Figure 6:**
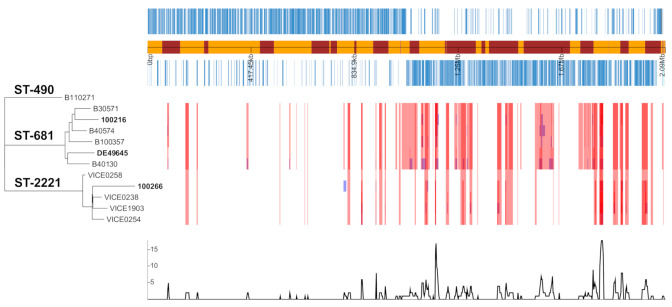
Maximum likelihood (ML) phylogeny and recombination events within serotype 6F and closely related 6A strains. On the left, is a ML phylogeny tree, constructed using only vertically inherited SNPs of the analyzed strains after masking recombination regions. Adjacent to the phylogeny reconstruction are blocks of red and blue depicting shared and unique homologous recombination events predicted by Gubbins, respectively. Underneath the blocks is a line graph summarizing total recombination hotspots along the genome. In total, there were 14,770 SNPs, of which 12,867 (87%) were introduced by 274 recombination events (Supplementary Materials Table S4).

As recombination tends to blur phylogenetic signals reflecting vertically inherited point mutations shared by common descent, we attempted to identify the true evolutionary history of the 6F genomes by masking any additional recombination regions within the whole-genome dataset alignments. The resulting maximum likelihood (ML) tree (Figure 6) rooted on the longest branch (outgroup strain) formed two distinct monophyletic lineages, which separated strains of the same sequence type (ST). As shown in Figure 6, the two Swiss 6F genomes clustered into different lineages based on ST: (1) Swiss 6F strain 100216 with German 6F strain DE49645 and other 6A strains of ST-681, and (2) Swiss 6F strain 1002.66 with other 6A strains of ST-2221. This separate cladistic clustering of each 6F strain shows that aside from expressing the same serotype, the three 6F strains shared higher identities and recombinational events with other 6A strains than among themselves.

Scrutinizing our phylogenetic analyses, two explanations are plausible to explain the evolution of the 6F strains. First, the SNP A150T leading to the evolution of the 6F serotype strains (Supplementary Materials Figure S3) has independently occurred twice, once in each of the two lineages with serotype 6A strains. Second, the SNP A150T has arisen once and then a capsular switch transferred the 6F capsule operon to the other clonal lineage. Based on our analyses which demonstrate more vertical SNPs outside than within the capsule region (Supplementary Materials Figure S4), we assume that the evolutionary event was rather a capsule switch than two independent SNP events for 1002.66. Given our limited sample size, this remains a hypothesis. although capsule switch events are known to be very frequent [22].

## 3. Discussion

The polysaccharide capsule of *S. pneumoniae* is the major virulence factor and is targeted by the current pneumococcal conjugate vaccines. Thus far, it has been broadly accepted that the structure of the capsule of *S. pneumoniae* is largely independent of the environment and is exclusively determined by the genes of the capsule operon [23]. This knowledge is important as it facilitates the creation and design of the PCVs and allows the exact assignation of serotypes in diagnostic laboratories using standard methods such as the Quellung reaction. Within this study, we have investigated three *S. pneumoniae* strains with serotype 6F for a possible dependency of the chemical structure of their capsules on the environment. We found that glucose is increasingly incorporated into the capsule when these strains are grown in CDM containing glucose as compared to galactose. This may be due to the increased availability of the capsule precursor UDP-glucose over UDP-galactose when grown in the different media. Using mAbs for serotype 6A and 6C, we further received a varying cross-reactivity depending on the composition of the growth medium. Finally, by using whole-genome sequencing, we found that there are at least three different serotype 6F strains found in Switzerland and Germany and that more than one evolutionary event took place that led to their emergence.

It has been previously shown that different pneumococcal isolates grow to different densities in some growth media [24]. The utilization of chemically defined medium (CDM) enables the evaluation of different environmental and nutritional factors on growth and fermentation patterns of *S. pneumoniae* under controlled conditions of pH, temperature and gas atmosphere [25]. In our study, we speculated that growth in CDM with different carbon sources may affect *S. pneumoniae* strains with bispecific glycosyltransferase. In more detail, an earlier study described that a mutation within WciNα changes the galactosyltransferase to a bispecific glycosyltransferase and created a new hybrid serotype 6F. Therefore, we aimed at investigating the strains of serotype 6F in different media [17]. Using defined growth conditions, we first found a better growth for glucose as compared to galactose for 6F strains which is common for *S. pneumoniae* as glucose is the preferred carbon source because of its quicker metabolization [18,19]. In addition, one of three strains showed a delayed growth and SNPs within genes with putative galactose metabolic function were identified. As we did not further investigate follow the SNPs, the mechanism for this phenomenon therefore remains unknown. The most notable finding of this study was the discovery of a significant decrease in the proportion of Glc’ incorporated into the polysaccharide capsule of serotype 6F strains when the strains were grown on galactose as the sole carbon source. UDP-glucose and UDP-galactose are the relevant capsule precursors for the incorporation of glucose and galactose into the capsule, respectively and we therefore measured these metabolites with ^31^P-NMR [26]. As expected, the amounts of precursors were very low for the wild-type strains as these metabolites are quickly converted. However, based on our results having increased UDP-glucose in glucose as compared to galactose for the knockout strain (106.66 dcps; Figure 4A,B), we hypothesize that when grown in glucose as the sole carbon source, an increased synthesis of UDP-glucose results in the incorporation of more glucose into the polysaccharide capsule by the bispecific glycosyltransferase. Similarly, UDP-glucose accumulated more in *S. pneumoniae* D39 of serotype 2 grown in glucose than in those grown in galactose [26]. However, there seems to be a sophisticated equilibrium as UDP-galactose can be either incorporated into the capsule or quickly epimerized to UDP-glucose by Gal-E (Figure 1). This feature of Gal-E may explain why we did not find a different ratio of UDP-glucose/UDP-galactose when strains were grown in the different media (Figure 4C).

We did not analyze the 6F strains when grown on a mixed (glucose-galactose) carbon substrate at early and late time points. However, it has been recently shown that glucose is always the preferred carbon source and that glucose and galactose are not metabolized in parallel [19]. We therefore speculate that 6F isolates alter their capsule after the glucose has been used and galactose is starting to get metabolized.

The three 6F strains in this study have been isolated in the period 2011–2014 and based on our whole-genome sequencing data, we hypothesize that the serotype 6F evolved from serotype 6A strains. Unlike serotype 6F, serotype 6A is included in PCV13 which was introduced in Switzerland in 2010 [4]. It is therefore tempting to speculate that the 6F strains evolved due to a competitive advantage towards the PCVs, especially considering that 6F strains may show a more 6C-like capsular composition in a glucose-rich environment (e.g., blood). Of course, despite presenting three 6F strains from two different countries in this study, the overall prevalence of 6F strains in the clinical setting is still very low. Possible reasons for this could be that the strains can be easily misclassified during conventional serotyping or that 6F strains have a competitive disadvantage compared to more relevant escape mutants, which would be serotype 6C in this case [27].

The human nasopharynx is the natural reservoir of *S. pneumoniae*. Mucin of the respiratory tract (e.g., of the nasopharynx) is generally composed of different sugars including galactose, mannose and N-acetylglucosamine but very little glucose [9,18]. In contrast to growth on mannose and N-acetylglucosamine, it has been shown that *S. pneumoniae* grown on galactose re-route their metabolic pathway from homolactic fermentation to a truly mixed acid fermentation regime [18]. In contrast to the respiratory tract, higher concentrations of glucose are found in the blood. Knowing whether the capsule structure of *S. pneumoniae* is dependent on a glucose/galactose carbon source may therefore be important for evaluating vaccine efficacy and future vaccine design. Notably, we do not report immunogenicity data but only show varying binding frequency of mAbs using an in vitro design in this study. Importantly, the inability to clearly define a serotype when the strains contain a bispecific glycosyltransferase may also be relevant during routine serotyping methods such as the Quellung reaction for epidemiological analysis [6].

A limitation of this study is that we do not show data as to whether there are any differences in competitive advantage, infectivity and survival in the host from strains 6F grown in glucose versus galactose in this study. We speculate that 6F strains are better protected as compared to serotype 6A from PCV13 (which includes 6A but not 6C). This is difficult to address as we do not know the exact composition of 6F at the different niches in the host. We again speculate that the Glc’ incorporation is increased in strains from blood, as blood is considered being glucose rich. However, this is challenging to address as NMR measurements need a high concentration of capsule from the bacteria which is difficult to receive directly from the host.

In summary, we report that the capsule structure for *S. pneumoniae* may indeed be dependent on the environmental composition of the carbon sources. We hypothesize that the dependence on carbon source is unique for serotypes with bispecific glycosyltransferase such as serotype 6F, as we did not observe structural differences for the other serogroup 6 strains (such as serotype 6A, 6B and 6C). As some niches of the body provide more galactose (e.g., the nasopharynx) than glucose (e.g., blood), 6F may display more galactose incorporation in a specific human niche. The prevalence of serotypes with bispecific glycosyltransferase seems to be currently low but we show that they have occurred in at least two countries, Switzerland and Germany.

## 4. Materials and Methods

### 4.1. Selection of Bacterial Isolates

Two invasive pneumococcal strains (1002.16 & 1002.66) isolated from blood, and observed in the Quellung reaction to react positively for both 6A and 6C sera (indicative for serotype 6F), were recovered from the bacterial strain collection of the National Center for Invasive Pneumococci (NZPn) at the Institute for Infectious Diseases (IFIK, Bern, Switzerland). As the isolates represented multi-locus sequence types (STs) 681 and 2221, respectively, further sampling was performed to include five additional invasive pneumococcal isolates from blood. All 5 isolates were randomly chosen, expressed serotype 6A antigenic profile and had 100% identical ST or sharing at least 6 of 7 alleles (single locus variant (SLV)) to the aforementioned 6F isolates. For reference comparison, the rare 6F German isolate (DE49645, ST681) originating from the German National Reference Center for Streptococci (NRCS, Aachen, Germany) was included. As ST-2221 was underrepresented in the selection, whole-genome sequencing (WGS) data from four blood isolates of ST2221 from Iceland [20] was retrieved from the curated PubMLST database [28] and included in the study analyses. All isolates used in this study are listed in Table 1.

### 4.2. Bacterial Growth Conditions

Bacteria were cultured as described previously [12]. Briefly, bacteria were streaked out on Columbia sheep blood agar (CSBA) plates and grown for ~10 h at 37 °C in a 5% CO_2_ atmosphere. They were then inoculated into tubes containing modified Lacks medium [29,30,31] supplemented with glucose or galactose and grown to an OD_600nm_ of 0.5. After centrifugation and washing, 3 mL of bacterial suspension at OD_600nm_ 0.5 were added to 150 mL of chemically defined medium (CDM) supplemented with a single carbon source at a concentration of 5.5 mM. The composition of the CDM can be found in the Supplementary Materials (Supplementary Materials Figure S5). The cultures were then grown further to mid–logarithmic phase. Bacterial growth was tracked by measuring the optical density at a wavelength of 600 nm (OD_600nm_) using a Thermo Scientific Helios Epsilon UV-vis spectrophotometer with an adapter to allow measurement of OD_600nm_ directly in the culture tubes.

### 4.3. Capsular Polysaccharide Extraction

Extracts of the capsular polysaccharide were obtained as described previously [12,32]. Bacterial cultures were grown as described above, harvested by centrifugation and washed with ice-cold H_2_O. After resuspension, capsule polysaccharide was separated from the cells by addition of buffer-saturated phenol to a concentration of 1% and incubation over night at room temperature. Cell debris was removed by centrifugation and nucleotides and peptides were digested by addition of nuclease and proteinase K, respectively. The capsule (cps) was separated from smaller molecules using Millipore Amicon Ultra 30 kDa cut off membrane centrifugal filter units and the solvent was removed under reduced pressure. Dried capsule polysaccharide samples were dissolved in 100 µL of D_2_O, transferred into 1.7 mm NMR micro tubes and submitted for NMR measurements.

### 4.4. H NMR Measurements of the Polysaccharide Capsule

NMR data were collected on a Bruker Avance II (500 MHz; ^1^H) spectrometer equipped with a 1.7 mm triple-resonance (^1^H, ^13^C, ^31^P) microprobe head or an inverse broadband (^1^H, X), 5 mm probe head. The samples were prepared as follows: The full amount of each capsule extract (~5–10 mg) was dissolved in 100 µL of pure D_2_O and 65 µL of the resulting mixtures was transferred into 1.7 mm NMR tubes. The dried extracellular samples were dissolved in 1 mL of D_2_O containing 10 mmol/L of TSP for quantification and 500 µL of the resulting mixtures was transferred into 5 mm NMR tubes. ^1^H spectra were recorded using 1024 scans, with a spectral width of 12,500.0 Hz, a recycling delay of 1 s and an acquisition time of 1.311 s. All spectra were acquired at a regulated temperature of 298 K. All experiments were recorded using the TopSpin^®^ software, version 3.2 (Bruker Biospin) and processed using TopSpin^®^ version 4.0.7. Unpaired p-tests have been used to compare the Glc’/Gal ratios of 6F grown in glucose as compared to galactose. Separate testing has been performed for early and mid-log phase.

### 4.5. Intracellular Metabolite Extraction, Identification and Quantification of Capsule Precursor Signals by ^31^P NMR

Intracellular metabolite extraction was performed as previously described [21]. In brief, the bacteria cultured in chemically defined medium were harvested, washed, resuspended in ice-cold H_2_O and diluted with absolute ethanol (EtOH) at −20 °C to a concentration of 60% EtOH. Cells were disrupted, cell debris was removed and the solvent was evaporated under reduced pressure. Dried samples were weighed and dissolved in 100 µL of NMR buffer (20 mM MOPS, 5 mM NaOAc, and 1 mM Ethylendiamintetraacetat (EDTA) in D_2_O with 0.1% phosphonoacetic acid (PPA), and 0.1% TSP, pH 7.4) transferred into 1.7 mm NMR microtubes and submitted for measurement of ^31^P NMR spectra. Quantification of the capsule precursor UDP-glucose and UDP-galactose was achieved by comparison to the quantity of the internal PPA standard following a classical calibration procedure. A specific conversion factor was obtained from this calibration and was used to calculate absolute amounts from the NMR integrals of UDP-glucose and UDP-galactose and PPA.

### 4.6. Fluorescence-Activated Cell Sorting (FACS) Experiments

Fixed bacterial aliquots were washed and normalized to OD600 of 0.02 in FACS buffer (PBS containing 4% fetal bovine serum (Thermo Scientific Hyclone, Logan, UT, USA), added to a V-bottomed ELISA plate (Sigma-Aldrich, St. Louis, MO, USA) and incubated with a culture supernatant of hybridoma, final dilution 1:40. For immunological comparison, all the strains were stained with a 6A (Hyp6AG4) or 6C (Hyp6DM5)-specific mAbs. Plates were incubated for 30 min at 4 °C, washed with FACS buffer and incubated again for 30 min at 4 °C with phycoerythrin-conjugated goat anti-mouse IgG antibody (1:1000). Bacteria were washed again, resuspended in FACS buffer and examined with a flow cytometer (Cytoflex, Beckman Coulter, Brea, CA, USA). Obtained data were analyzed with FlowJo^TM^ 10. Unpaired p-tests have been used to compare the labelling of 6F grown in glucose as compared to galactose (see text for details).

### 4.7. DNA Library Preparation and Whole-Genome Sequencing (WGS)

DNA was extracted using QIAamp DNA mini kit (Qiagen, Hilden, Germany) and subsequently purified with the QIAcube^®^ automation station (Qiagen, Germany). WGS was performed at Eurofins Genomics Europe Sequencing GmbH (Konstanz, Germany) with Illumina NovaSeq 6000 system (Illumina, CA, USA) using paired-end 150 base-pair read length.

For the purpose of generating comparable, high-quality, circularized and complete genomes via hybrid assembly, Oxford Nanopore Technology (ONT) GridION sequencing was additionally performed for the three 6F isolates: 1002.16, 1002.66 and DE49645. Libraries were prepared with the 1D^2^ sequencing kit (SQK-LSK309) according to the manufacturer’s instructions and nanopore sequencing was performed with one isolate per R9.5.1 flow cell on GridION.

### 4.8. Genomic Analysis and Assembly

Genomic analyses, assembly creations, genome comparison and pan-genome analyses were conducted as described in supplemental material. To assess for genome composition variations within the serotype 6F and their closely related 6A strains, BLAST comparisons of each assembled genome against the hybrid-assembled DE49645 genome were performed. Whole-genome comparison and Pan-Genome Analysis were subsequently performed (Supplementary Materials).

### 4.9. Phylogenetic and Recombination Analysis

To investigate the evolutionary relatedness of the Swiss 6F isolates to the German 6F isolate, in the context of their closely related 6A isolates, whole-genome sequence alignment of all study isolates was first generated with Snippy v4.3.6 [33]. In brief, sequence reads of all strains were aligned using the outlier strain, B11072-51 of ST-490, as reference. SNPs within core genome loci (sites with base calls for all isolates) of the whole-genome alignment were filtered to only include high-quality SNPs (minimum coverage of 15X, minimum alternate allele frequency of 0.99 and minimum base quality of 20).

Snippy generated a 2.02 Mb whole-genome alignment of all 12 genomes and identified a total of 7684 high-quality, informative, core SNP sites. Heterogeneous (HET), low coverage (LC) and unaligned regions were assessed for each genome and at the maximum, only accounted for less than 0.04%, 0.4% and 3%, respectively of genome assemblies. Values for HET and LC regions reflected minimal genome fractions and were therefore considered negligible sources of genetic information for phylogenetic analysis. Unaligned sequences were suspected to have been imported from external sources via recombination and were verified by using Basic Local Alignment Search Tool (BLAST) of their draft assemblies against the NCBI nucleotide database accordingly. Top hits for the largest contig of each assembled unaligned sequence had high identities to pneumococcal prophages, confirming recombination suspicions and justifying variant filtering parameters as suitable and non-stringent.

Gubbins software v2.3.4 [34] and the RAxML model were then used to produce a maximum likelihood (ML) phylogeny, accounting for putative recombination events while reconstructing recent diversification of the strains from their most recent common ancestor (MRCA). The ML phylogeny was outgroup rooted using isolate B11072-51 of ST-490 as outgroup. This isolate expresses serotype 6A and is a SLV to the two main sequence types (ST-681 & ST-2221) in this study. Resulting recombination predictions from Gubbins were visualized using Phandango [35].

Illumina and nanopore WGS reads for the hybrid assembled isolates 1002.16, 1002.66 and DE49645 were deposited in the NCBI Sequence Read Archive (SRA) under project accession PRJNA625550 (See Table 1).

## Figures and Tables

**Table 1:** Details of serogroup 6 *S. pneumoniae* strains used in this study.

	MLST	Serotype	Year of Isolation	Isolate	Country	Genome Size (bp)	Reference Accession Number
B100357	681	6A	2011	Blood	Switzerland	2,159,962 **	This study; SRR11550454
B110271	490	6A	2011	Blood	Switzerland	2,091,771 **	This study; SRR11550453
B30571	681	6A	2004	Blood	Switzerland	2,141,354 **	This study; SRR11550452
B40130	681	6A	2005	Blood	Switzerland	2,116,757 **	This study; SRR11550451
B40574	681	6A	2005	Blood	Switzerland	2,167,664 **	This study; SRR11550459
VICE0238 *	2221	6A	2011	Blood	Iceland	2,132,191 **	[20]; ERR388909
VICE0258 *	2221	6A	2012	Blood	Iceland	2,130,915 **	[20]; ERR388920
VICE0254 *	2221	6A	2012	Blood	Iceland	2,132,423 **	[20]; ERR388919
VICE1903 *	2221	6A	2014	Blood	Iceland	2,137,293 **	[20]; ENA: ERR755620
106.66	2244	6B		NP	Switzerland	2,130,915 **	[21]; ENA: ERR388920
106.66 *Δcps* Janus	2244	nt		NP	Switzerland	n.a.	[21]
203.24	n.a.	6C		NP	Switzerland	n.a.	[21]
1002.16	681	6F	2010	Blood	Switzerland	2,238,890 ***	This study; SRR9943963; SRR9960027
1002.66	2221	6F	2011	Blood	Switzerland	2,184,396 ***	This study; SRR9943964; SRR9960028
DE49645	681	6F	2012	Blood	Germany	2,151,471 ***	[17]; SRR11550458; SRR11550455

* Only the whole-genome sequencing data from the Icelandic isolates were available and used in this study. ** Based on Illumina sequencing only. *** The 6F strains have two SRA accession numbers corresponding to Illumina reads and Nanopore (GridION) reads, respectively.

## Data Availability

Illumina and nanopore WGS reads for the hybrid assembled isolates 1002.16, 1002.66 & DE49645 were deposited in the NCBI Sequence Read Archive (SRA) under project accession PRJNA625550.

## Supplementary Materials

The following are available online at https://www.mdpi.com/article/10.3390/ijms22094580/s1, Supplementary methods and results for the whole-genome sequencing analyses, Figure S1: ^1^H-NMR spectra of capsular polysaccharides of serogroup 6 strains, Figure S2: Comparative genomic analyses of pneumococcal serotype 6F and 6A genomes of the same MLST, Figure S3: SNPs in the capsule region, Figure S4: Density of vertical SNPs, and Figure S5: Recipe of CDM. Tables S1–S4.
