# Supplementary material for: Carbon Source-Dependent Changes of the Structure of Streptococcus pneumoniae Capsular Polysaccharide with Serotype 6F
Source: Int J Mol Sci. 2021 Apr 27;22(9):4580. doi: 10.3390/ijms22094580 (PMC8123889; doi:10.3390/ijms22094580)
Supplement: Supplementary file 1 [file ijms-22-04580-s001.zip › ijms-1179336-supplementary/supplementary_R1_vf/Supplementary_material_figures_R1_vf.pdf]

Supplementary methods and results for the whole genome sequencing analyses

Genomic analysis and Assembly

Quality assessment of Illumina raw sequencing data from all 12 isolates was done with FastQC v0.11.7 (1). For raw nanopore reads, adapters were trimmed off using Porechop v0.2.3 (2) and quality control metrics were generated with Nanoplot v1.0.0 (3). *In Silico* Multi Locus Sequence Typing (MLST) and serotyping were done to confirm the ST and serotypes of the quality-checked Illumina WGS reads using *string*MLST v0.5.1 (4) and SeroBA v1.0.0 (5) respectively. *De novo* assemblies for all five sequenced 6A isolates and the four PubMLST isolates were generated using the Illumina-only assembly mode of the Unicycler v0.4.7 pipeline (6) while the Hybrid assembly mode was used to generate complete genomes for the three 6F isolates. Finally, raw reads were mapped back onto assemblies of each isolate via Minimap2 v2.14-r883 (7) to verify SNP differences. *De novo* genome assembly generated sequences ranging in length from 2.09 Mb to 2.17 Mb (Table 1). The hybrid assembled genomes of the two Swiss 6F isolates were notably larger than that of their German counterpart by an increment of ~33kb (100266) and ~87kb (100216). This was paralleled with an increase in the number of Prokka annotated coding sequence (CDS) genes for the aforementioned Swiss genomes. The sequencing read coverage for the draft and complete genomes ranged from 75x to 354x. Coverage was unsurprisingly higher for the eight genomes that were freshly sequenced in this study (**Error! Reference source not found.**), as newer and higher throughput sequencing platforms (NovaSeq 6000 & GridION) were used compared to the already published PubMLST reads which were sequenced in previous years with older platforms (HiSeq 2000).

The Illumina and nanopore WGS reads for the hybrid assembled isolates 100216, 100266 & DE49645 were deposited in the NCBI Sequence Read Archive (SRA) under project accession PRJNA625550 (See Table 1).

Whole genome comparison and Pan-Genome Analysis

Assemblies were annotated using Prokka v1.14.0 (11). The genomic content of the draft and hybrid assemblies was examined using the BLAST Ring Image Generator (BRIG; v0.95) (9). BRIG was run with the BLASTn algorithm (10) and an *e*-value of 1e5 using DE49645 as reference. Regions showing high level of variability were noted and labelled accordingly (Supplementary figure 1).

Pan genome analysis was conducted using Roary v3.11.0 (12). COG (Clusters of Orthologous Groups of proteins) functional categories were assigned by blasting proteins against the EggN0G v5.0 database (13) using the online EggNOG-mapper v2 (14) with a minimum *e*-value of 0.001 and query coverage of 75%. A total of 2436 orthologous genes were identified, of which 1956 were conserved among all three 6F genomes and thereby defined as the core genome of all 6F strains. The remaining 480 orthologous genes were classified as accessory genome and divided into "Variable genes"; defined as those shared by two genomes and, "Unique genes"; defined as those present in only one genome. Unique genes accounted for less than 6% of the genome for all 6F strains with strain 100266 having the highest proportion (5.96%) and strain DE49645 having the lowest (1.89%).

References

1. Andrews S. 2010. FastQC: a quality control tool for high throughput sequence data, http://www.bioinformatics.babraham.ac.uk/projects/fastqc/.
2. Wick RR. 2017. Porechop: adapter trimmer for Oxford Nanopore reads, https://github.com/rrwick/Porechop.
3. De Coster W. 2017. NanoPlot: Plotting tool for long read sequencing data and alignments., https://github.com/wdecoster/NanoPlot.
4. Gupta A, Jordan IK, Rishishwar L. 2017. stringMLST: a fast k-mer based tool for multilocus sequence typing. Bioinformatics 33:119-121.
5. Epping L, van Tonder AJ, Gladstone RA, The Global Pneumococcal Sequencing C, Bentley SD, Page AJ, Keane JA. 2018. SeroBA: rapid high-throughput serotyping of Streptococcus pneumoniae from whole genome sequence data. Microb Genom 4.
6. Wick RR, Judd LM, Gorrie CL, Holt KE. 2017. Unicycler: Resolving bacterial genome assemblies from short and long sequencing reads. PLoS Comput Biol 13:e1005595.
7. Li H. 2018. Minimap2: pairwise alignment for nucleotide sequences. Bioinformatics 34:3094-3100.
8. Garcia-Alcalde F, Okonechnikov K, Carbonell J, Cruz LM, Gotz S, Tarazona S, Dopazo J, Meyer TF, Conesa A. 2012. Qualimap: evaluating next-generation sequencing alignment data. Bioinformatics 28:2678-9.
9. Alikhan NF, Petty NK, Ben Zakour NL, Beatson SA. 2011. BLAST Ring Image Generator (BRIG): simple prokaryote genome comparisons. BMC Genomics 12:402.
10. Altschul SF, Gish W, Miller W, Myers EW, Lipman DJ. 1990. Basic local alignment search tool. J Mol Biol 215:403-10.
11. Seemann T. 2014. Prokka: rapid prokaryotic genome annotation. Bioinformatics 30:2068-9.
12. Page AJ, Cummins CA, Hunt M, Wong VK, Reuter S, Holden MT, Fookes M, Falush D, Keane JA, Parkhill J. 2015. Roary: rapid large-scale prokaryote pan genome analysis. Bioinformatics 31:3691-3.
13. Huerta-Cepas J, Szklarczyk D, Heller D, Hernandez-Plaza A, Forslund SK, Cook H, Mende DR, Letunic I, Rattei T, Jensen LJ, von Mering C, Bork P. 2019. eggNOG 5.0: a hierarchical, functionally and phylogenetically annotated orthology resource based on 5090 organisms and 2502 viruses. Nucleic Acids Res 47:D309-D314.
14. Huerta-Cepas J, Forslund K, Coelho LP, Szklarczyk D, Jensen LJ, von Mering C, Bork P. 2017. Fast Genome-Wide Functional Annotation through Orthology Assignment by eggNOG-Mapper. Mol Biol Evol 34:2115-2122.

Figure Legends

Supplementary figure 1: ^{1}H-NMR spectra of capsular polysaccharides of sergroup 6 strains. Complete 1D ^{1}H-NMR spectra were obtained for different serogroup 6 strains.

Supplementary figure 2: Comparative genomic analyses of pneumococcal serotype 6F and 6A genomes of the same MLST.

BLAST comparison of two newly discovered pneumococcal serotype 6F genomes from Switzerland and closely related 6A genomes against a reference (German 6F strain DE49645) via the Blast Ring Image Generator (BRIG). Gaps in rings reflect regions with less than 70% identity to the reference. Nine regions of variability (depicted by dotted boxes) show major areas of genetic differences between the German serotype 6F genome and the two Swiss 6F genomes. The three innermost rings represent the three serotype 6F genomes and are separated by a white space from genomes having serotype 6A. Isolates with the same MLST sequence are illustrated in decreasing colour intensity (dark navy blue to sky blue for ST681, reddish peach to light peach for ST2221 and green for ST490) starting from the three 6F genomes of the innermost rings and ending at the four Icelandic genomes of serotype 6A.

Supplementary figure 3. SNPs in the capsule region. Nucleotide substitutions in the capsular region and flanking genes of the serotype 6F and 6A strains.

Supplementary figure 4. Density of vertical SNPs. The density of vertical SNPs of (A) 1002.16 compared to 1002.66 and (B) DE49645 compared to 1002.66 are shown. Vertical SNPs were calculated per 15 kb and mean is indicated by a dashed horizontal red line while the capsule region is denoted by dashed vertical red lines.

Supplementary figure 5. Recipe of Chemically defined medium (CDM)

Supplementary figure 1

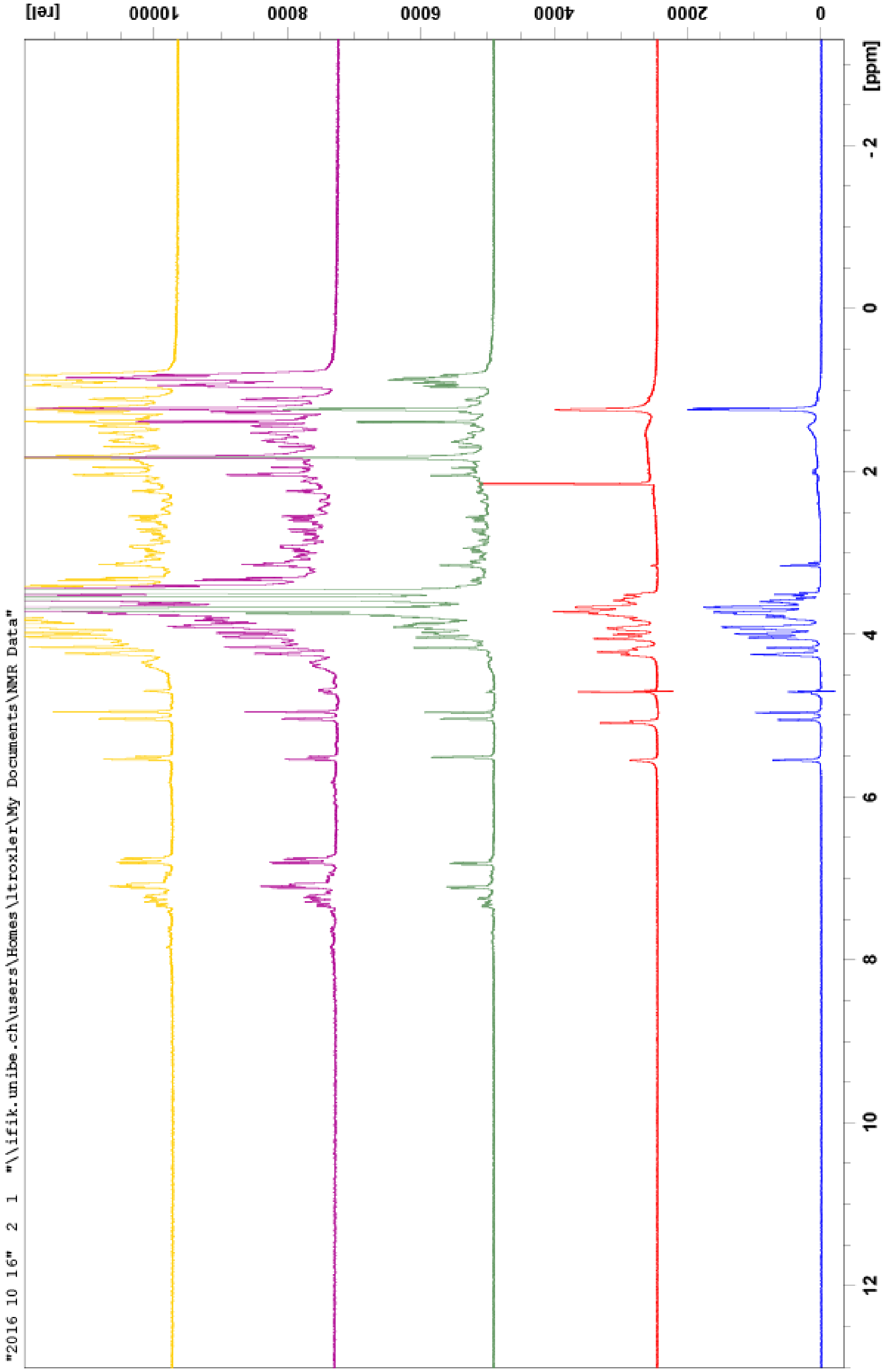

Supplementary figure 2

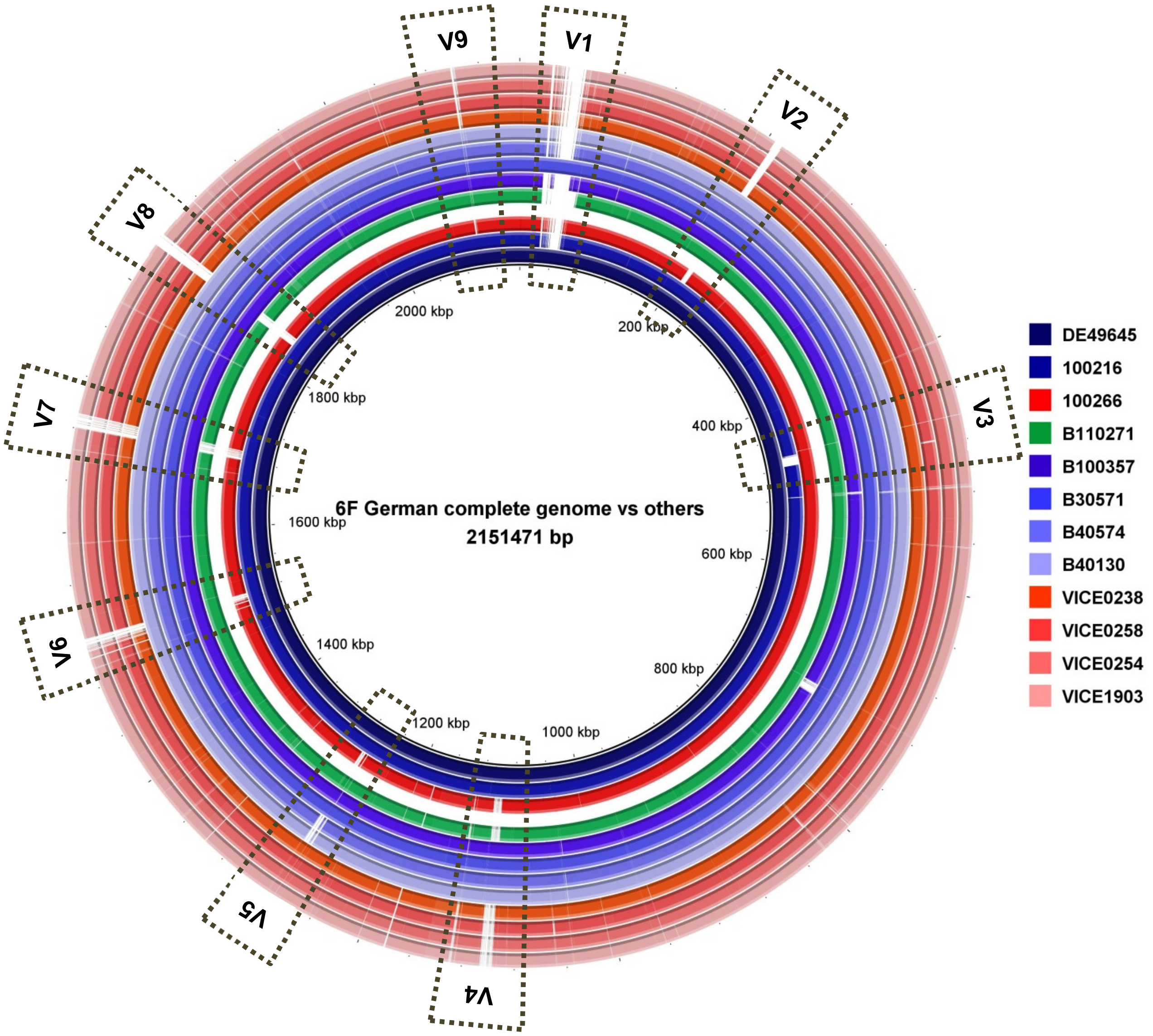

Supplementary figure 3

6F SNP (arrow pointing down to the *wciN* column, position 16642)

	dexB	intR upstream *wzg*	*wzd*	*wze*		*wchA*	intR upstream *wciN*	*wciN*	*wzy*	*glf_1*			
		1	1	1	1	1	1	1	1	2	2		
	8	1	3	4	4	4	6	6	9	6	6		
	6	1	5	4	6	8	1	6	3	0	1		
	2	1	1	8	1	6	2	4	3	2	6		
	6	6	9	4	0	4	5	2	9	0	4		
B110271 fullcapsule flank:pbp2x-pbp1a	c	a	c	a	c	c	a	g	g	c	t	6A	ST490
VICE0254 fullcapsule unicycler:pbp2x-pbp1a	.	t	.	.	.	.	g	.	t	.	.	6A	ST2221
VICE1903 fullcapsule unicycler:pbp2x-pbp1a	t	t	.	.	.	t	g	.	.	.	.	6A	ST2221
VICE0258 fullcapsule unicycler:pbp2x-pbp1a	.	t	.	.	.	.	g	.	.	.	.	6A	ST2221
VICE0238 fullcapsule unicycler:pbp2x-pbp1a	.	t	.	g	.	.	g	.	.	.	.	6A	ST2221
100266 full capsule flanks:pbp2x-pbp1a	.	t	.	.	.	.	g	a	.	.	.	6F	ST2221
DE49645 fullcapsule flanks:pbp2x-pbp1a	.	t	.	.	.	.	g	a	.	.	.	6F	ST681
100216 full capsule flanks: pbp2x-pbp1a	.	t	.	.	.	.	g	a	.	.	.	6F	ST681
B100357 fullcapsule flank:pbp2x-pbp1a	.	t	.	.	t	.	g	.	.	t	.	6A	ST681
B40574 fullcapsule flanks:pbp2x-pbp1a	.	t	.	.	.	.	g	.	.	.	a	6A	ST681
B40130 fullcapsule flank:pbp2x-pbp1a	.	t	t	.	.	.	g	.	.	.	.	6A	ST681
B30571 fullcapsule flank:pbp2x-pbp1a	.	t	.	.	.	.	g	.	.	.	.	6A	ST681

Supplementary figure 4

Supplementary figure 4A: Density of 100216 vertical SNPs (REF−100266)

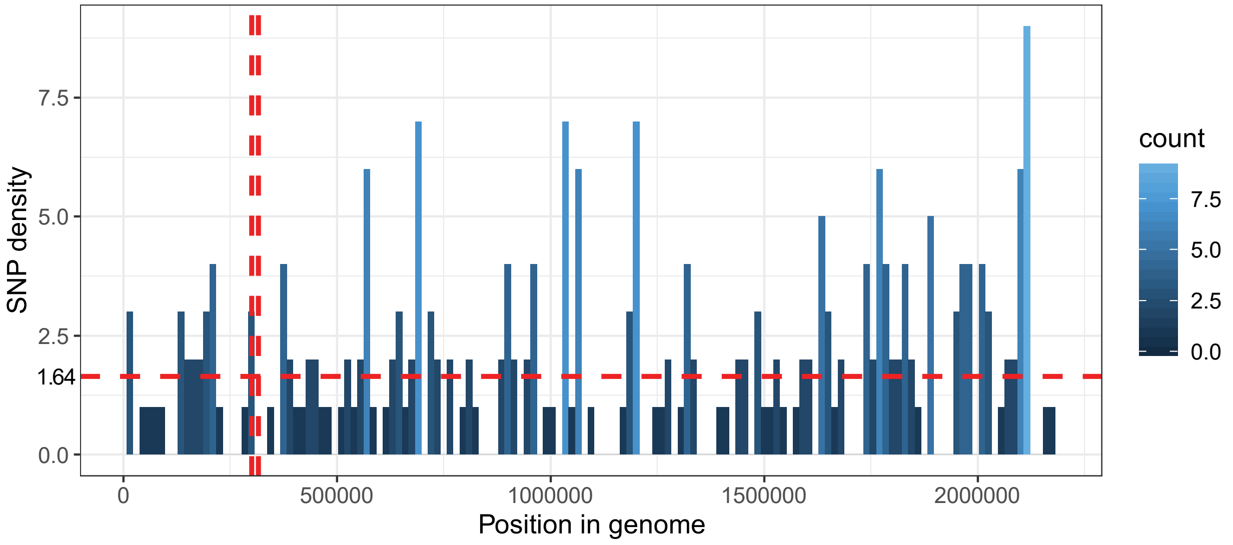

Supplementary figure 4B: Density of DE49645_6F vertical SNPs (REF−100266)

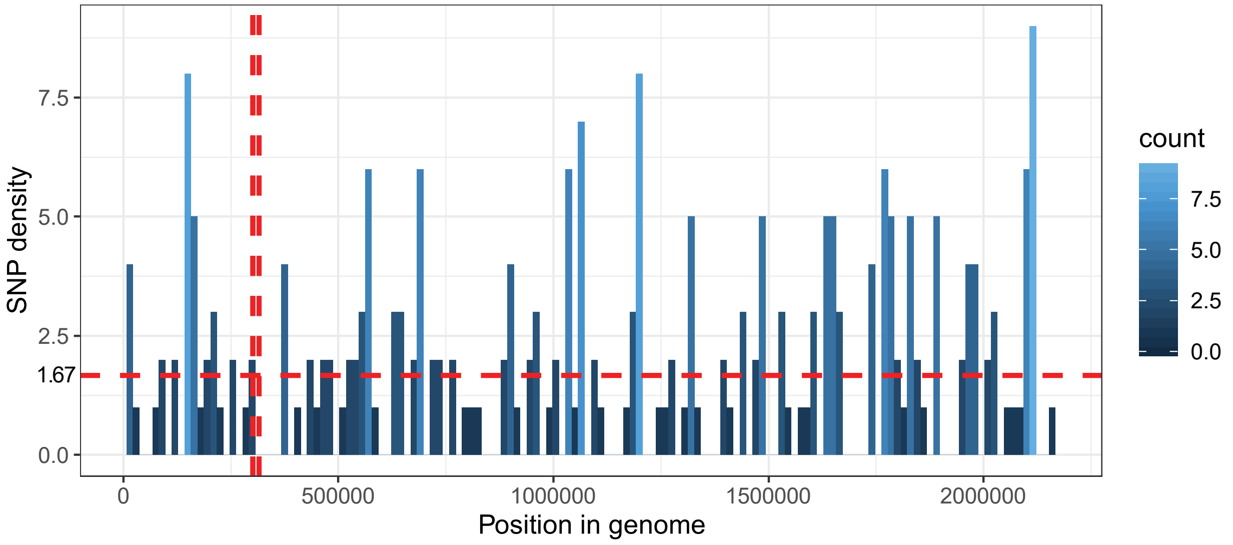

Supplementary figure 5

Component	Concn (mg/liter)
1. $FeSO_4 \cdot 7H_2O$	5
$Fe(NO_3)_2 \cdot 9H_2O$	1
K_2HPO_4	200
KH_2PO_4	1,000
$MgSO_4 \cdot 7H_2O$	700
$MnSO_4$	5
2. DL-Alanine	100
L-Arginine	100
L-Aspartic acid	100
L-Cystine	50
L-Glutamic acid	100
L-Glutamine	200
Glycine	100
L-Histidine	100
L-Isoleucine	100
L-Leucine	100
L-Lysine	100
L-Methionine	100
L-Phenylalanine	100
L-Proline	100
Hydroxy-L-proline	100
L-Serine	100
L-Threonine	200
L-Tryptophan	100
L-Tyrosine	100
L-Valine	100
3. *p*-Aminobenzoic acid	0.2
Biotin	0.2
Folic acid	0.8
Niacinamide	1
β-Nicotinamide adenine dinucleotide	2.5
Pantothenate calcium salt	2
Pyridoxal	1
Pyridoxamine dihydrochloride	1
Riboflavin	2
Thiamine hydrochloride	1
Vitamin B_{12}	0.1
4. Glucose	10,000
5. Adenine	20
Guanine hydrochloride	20
Uracil	20
6. $CaCl_2 \cdot 6H_2O$[a]	10
$NaC_2H_3O_2 \cdot 3H_2O$	4,500
L-Cysteine	500
$NaHCO_3$	2,500
$NaH_2PO_4 \cdot H_2O$	3,195
Na_2HPO_4	7,350

[a] BDH Chemicals, Ltd., analytical reagent.

Modifications for study

+ 15.4 g/l MES (2-(N-morpholino) ethanesulfonic acid)

K_2HPO_4 removed completely

KH_2PO_4 reduced to 200 mg/l
